# The complete genome sequence of *Sorbus insignis* (Rosaceae: Amygdaloideae), an epiphytic shrub in this genus

**DOI:** 10.1080/23802359.2020.1810150

**Published:** 2020-08-26

**Authors:** Jing Tan, Yi Wang, Wei Wu, Yongquan Li, Wanyi Zhao, Wenbo Liao, Qiang Fan, Boyong Liao

**Affiliations:** a College of Horticulture and Landscape Architecture, Zhongkai University of Agriculture and Engineering, Guangzhou, Guangdong, China; b State Key Laboratory of Bio-control and Guangdong Provincial Key Laboratory of Plant Resources, Sun Yat-Sen University, Guangzhou, China

**Keywords:** *Sorbus insignis*, Rosaceae, chloroplast genome, illumina sequencing

## Abstract

*Sorbus insignis* (Hook. f.) Hedl., belonging to *S. sect. Multijugae* Yu is an epiphytic shrub. Its phylogenetic position is still poorly understood. In this study, we assembled its complete chloroplast genome from whole-genome high-throughput sequencing data. The chloroplast genome was 159, 993 bp in length, with a large single-copy (LSC) region of 87, 932 bp, a small single-copy (SSC) region of 19, 255 bp, separated by two inverted repeat (IR) regions of 26, 403 bp each. It was predicted to contain a total of 132 genes, with an overall GC content of 36.56%. Phylogenetic analysis suggested *S. insignis* belongs to *S.* L. *sensu stricto* and closest to *S. prattii* Koehne among the published chloroplast genome.

*Sorbus insignis* (Hook. f.) Hedl., belonging to *S. sect. Multijugae* Yu (Rosaceae) is a shrub native to the southwest of China, north of Myanmar, north of India, and Nepal (Yu and Lu 1997; Lu et al. 2003). This species is quite distinct in genus *Sorbus*, which occurs epiphytic habits usually climbing along the trunk and branch of broadleaf trees or cliff and rocks, and distributed at an altitude from 2600 to 3300 m. The morphological and systematics studies indicated *S. insignis* is most closely related to *S. Harrowiana* (Balf. F. et W. W. Smith) Rehd., a species character in larger leaf and distributed along Gaoligong Mountain (Yu et al. 1997; Lu and Stephen 2003; Lo and Donoghue 2012; Li et al. 2017). While, some flora and taxonomy study treated the *S. harrowiana* as a synonym of *S. insignis* (Lu and Stephen 2003). This taxonomic confusion could be solved by phylogenetic studies. In this study, the complete chloroplast genome sequence of *S. insignis* was sequenced and characterized. We also constructed a phylogenetic tree to confirm its relationship with other species within the genus *Sorbus*. The annotated genome sequence is accessible from GenBank with the accession number (GenBank: MT677871).

Fresh leaves of *S. insignis* were sampled in Gaoligong Mountain National Nature Reserve, Lushui city, Yunnan Province, China (25.980275 N, 98.696044E). The voucher specimen (B.Y. Liao & W.Y. Zhao 2018084) was deposited in the Herbarium of Sun Yat-sen University (SYS). Total genomic DNA was extracted from silica-dried leaves using the modified CTAB method. The DNA library was prepared with a TruSeq DNA Sample Prep Kit (Illumina, USA) according to the instructions of the manufacturer. Then the DNA library was sequenced on an Illunima Hiseq 2500 Sequencing System (Guangzhou, China). A total of 6 Gb paired-end sequence (150 bp) was generated and used to assemble the chloroplast genome in GetOrganelle (Jin et al. 2018). The genome annotation was performed by the Geseq online tool (Tillich et al. 2017) and Geneious ver. 10.1 (Kearse et al. 2012), then manually verified and corrected by comparison with sequences of related species, for example, *S. tianschanica* (GenBank: MK920289), *S. prattii* (GenBank: MK814479), and *S. tianschanica* (GenBank: MK920289).

The circular chloroplast genome *S. insignis* was 159,993 bp in length, with a large single-copy (LSC) region of 87, 932 bp, a small single-copy (SSC) region of 19, 255 bp, separated by two inverted repeat (IR) regions of 26, 403 bp each. It was predicted to contain 132 genes, including 87 protein-coding genes, 37 tRNA genes, and 8 rRNA genes. The overall GC content was 36.56%.

To investigate the relationship of *S. insignis*, the chloroplast genomes of *S. insignis* and 16 other species were aligned using MAFFT ver. 7.307 (Katoh and Standley 2013), and the *Malus domestica* was selected as outgroup. A phylogenetic tree (Figure 1) was constructed with the maximum likelihood method using RAxML (Stamatakis 2014). The result of the phylogenetic analysis revealed that *Sorbus* L. *sensu lato* is not monophyletic (Figure 1), which is consistent with previous research results (Lo and Donoghue 2012; Li et al. 2017). The *S. insignis* is nested within *S.* L. *sensu stricto* and sister to *S. prattii* Koehne and *S. setschwanensis* (Schneid.) Koehne. *S. insignis* were previously placed in *S.* Ser. *Insignes* Yu (Yu and Kuan 1963), while a molecular systematics bases on four nuclear and one chloroplast marker suggest to place it into *S.* Ser. *Multijugae* Yu (Li et al. 2017). Our results support *S. insignis* belongs to *S.* L. *sensu stricto*. The chloroplast genome of *S. insignis* reported here provides new resources for the phylogenetic study of the genus *Sorbus*.

**Figure 1.**
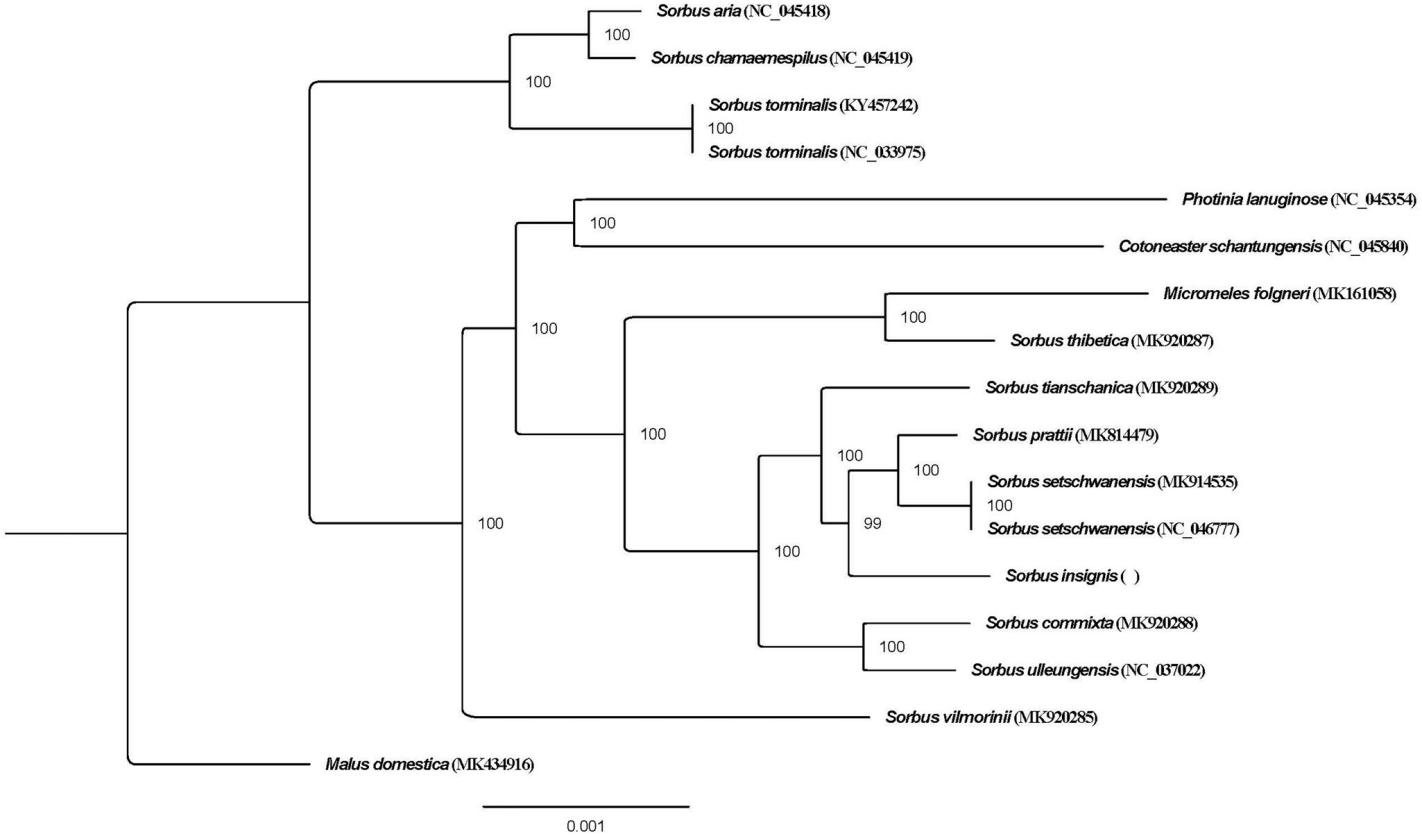
Maximum likelihood tree of genus *Sorbus* based on complete chloroplast genomes, with *Malus domestica* as outgroup. Bootstrap support values (based on 1000 replicates) are shown next to the nodes. Scale in substitutions per site.

## Data Availability

The data that support the findings of this study are openly available in GenBank of NCBI at https://www.ncbi.nlm.nih.gov, reference number MT677871.
